# Probiotic Human Study Design and Reporting (PRODARE): Recommendations to Improve Research Practice^☆^

**DOI:** 10.1016/j.advnut.2026.100668

**Published:** 2026-06-02

**Authors:** Hania Szajewska, Marc Benninga, Georgia Chatonidi, Adele Costabile, Jonathan Lane, Carolien A van Loo-Bouwman, Cath Mersh, Patricia Sanz Morales, Gemma Walton, Maria Carmen Collado, Ruggiero Francavilla, Christine Fulling, Benjamin Anderschou Holbech Jensen, David Perez Pascual, Paul R Ross, Chris HP van den Akker, Yvan Vandenplas, Nikoletta Vidra, Arthur C Ouwehand

**Affiliations:** 1 Department of Paediatrics, The Medical University of Warsaw, Warsaw, Poland; 2 Department of Paediatric Gastroenterology, Emma Children’s Hospital, Amsterdam University Medical Center, University of Amsterdam, Amsterdam, The Netherlands; 3 International Life Sciences Institute Europe, Brussels, Belgium; 4 School of Medical Sciences, Faculty of Medicine and Health, Nutrition-Gut-Brain Interactions Research Centre, Örebro University, Örebro, Sweden; 5 School of Life and Health Sciences, University of Roehampton, London, United Kingdom; 6 Health and Happiness (H & H) Group, H & H Research, National Food Innovation Hub, Teagasc Moorepark, Fermoy, Co. Cork, Ireland; 7 Yili Innovation Center Europe, Wageningen, The Netherlands; 8 Cath Mersh Communications, Aarhus, Denmark; 9 Department of Food and Nutritional Sciences, University of Reading, Whiteknights, Reading, United Kingdom; 10 Department of Biotechnology, Institute of Agrochemistry and Food Technology—National Research Council (IATA-CSIC), Valencia, Spain; 11 Interdisciplinary Department of Medicine, Paediatric Section, University of Bari Aldo Moro, Bari, Italy; 12 Cargill Research and Development Centre Europe BV, Vilvoorde, Belgium; 13 Department of Biomedical Sciences, Faculty of Health and Medical Sciences, University of Copenhagen, Copenhagen, Denmark; 14 Danone Global Research & Innovation Center, Gif-sur-Yvette, France; 15 Alimentary Pharmabiotic Centre Microbiome Ireland, University College Cork, Cork, Ireland; 16 Department of Pediatrics—Neonatology, Emma Children’s Hospital, Amsterdam UMC, Amsterdam Reproduction & Development Research Institute, University of Amsterdam, Amsterdam, The Netherlands; 17 KidZ Health Castle, UZ Brussel, Brussels, Belgium; 18 Science Department, Yakult Europe, Almere, The Netherlands; 19 Health R&D, International Flavors & Fragrances Inc Health, Kantvik, Finland

**Keywords:** probiotics, human study design, recommendations, exploratory studies, pilot studies, randomized controlled trials, real-world studies, observational cohort studies

## Abstract

The PRObiotic human study Design And REporting recommendations provide a structured framework for designing and reporting human studies with probiotics, addressing challenges related to product viability. Five main study types are identified. Exploratory studies are early-phase, first-in-human investigations designed to evaluate effects or generate hypotheses and require specification of strain, dose, stability, and assessment of acceptance and adverse events. Pilot studies assess the feasibility of protocols and logistics before larger trials, with clearly defined objectives, compliance measures, and data management plans. Randomized controlled trials represent the reference standard for evaluating efficacy and safety and include the use of core outcome sets, blinding procedures, and adherence monitoring. Real-world studies evaluate effectiveness outside controlled research settings and require detailed protocols, comprehensive data capture, and predefined approaches for handling inconsistencies. Observational cohort studies examine associations between probiotic exposure and outcomes over time, with clearly defined endpoints, cohort selection criteria, and analysis plans. Across all study types, probiotic-specific considerations include strain characterization and sequencing, verification of identity, purity, viability, antimicrobial resistance, minimization of confounding factors, management of cross-contamination, and consistency of dosage. These recommendations aim to enhance study quality and reporting and to guide researchers in selecting study designs appropriate to their objectives.

## Graphical abstract

**Figure:**
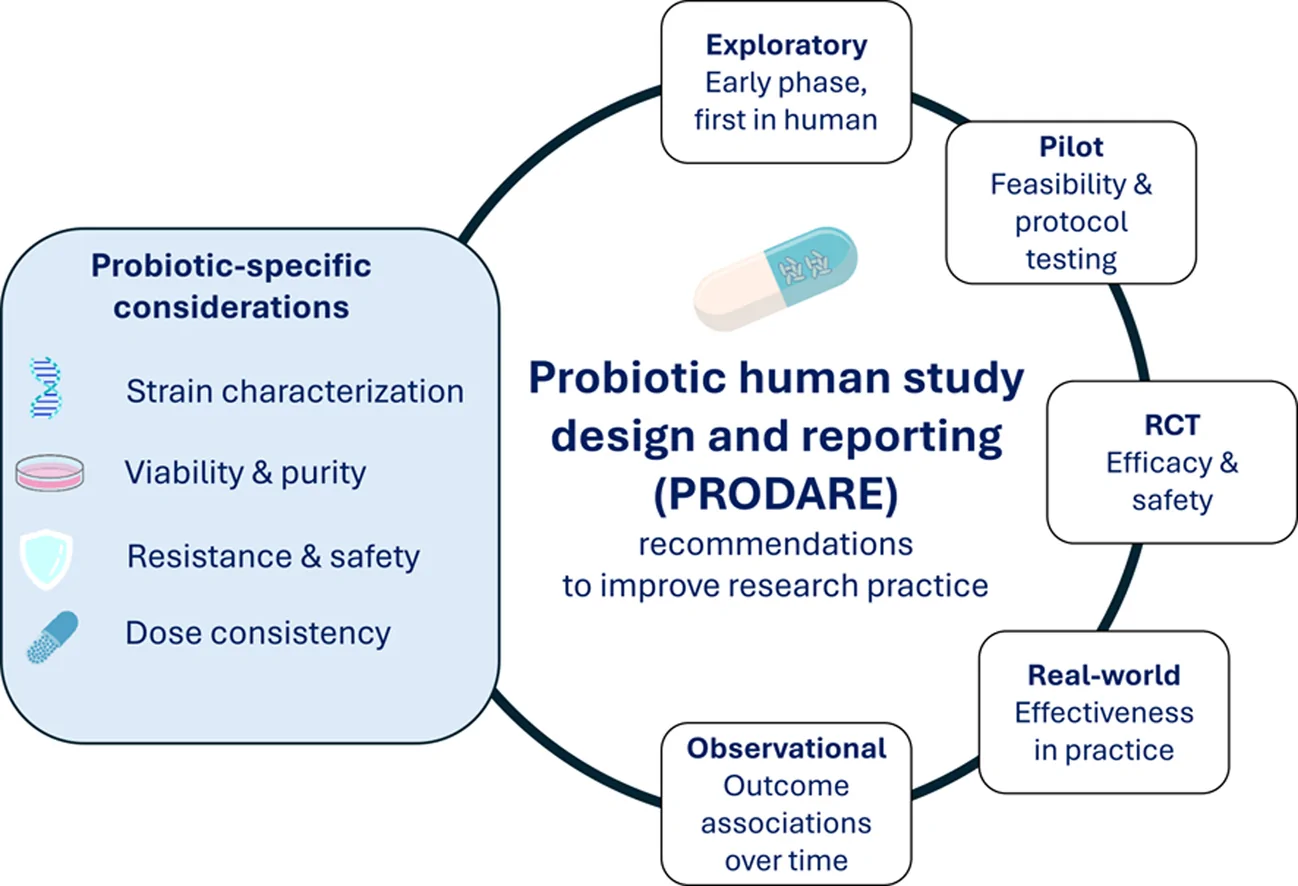

## Introduction

**Statement of Significance**

This work is the first to provide a dedicated, consensus-based framework (probiotic human study design and reporting) specifically tailored to the design and reporting of human probiotic studies, addressing methodological challenges unique to live, strain-specific microorganisms. It introduces a structured decision tree linking study objectives to appropriate designs and reporting standards, thereby extending existing generic guidelines with probiotic-specific recommendations.

Probiotics are defined as “live microorganisms that, when administered in adequate amounts, confer a health benefit on the host” [1]. Human studies with specific probiotic strains have demonstrated their potential to prevent or alleviate a range of illnesses and conditions [2].

Despite the expansion of probiotic research, inconsistencies in the design and reporting of probiotic human studies remain a significant impediment to advancing the field. This delays progress toward regulatory acceptance and the commercialization of well-documented probiotics for incorporation in food products and dietary supplements.

Although several established guidelines inform the conduct of human nutrition studies, these frameworks are predominantly generic and do not address the distinctive methodological complexities associated with probiotic research. Consequently, researchers may encounter ambiguity when selecting appropriate study designs or when interpreting and reporting findings in a way that accurately captures probiotic mechanisms of action and associated health outcomes. This lack of specificity compromises the reproducibility, comparability, and, ultimately, the scientific and regulatory legitimacy of study findings.

The Probiotics Task Force of International Life Sciences Institute (ILSI) Europe initiated a collaborative effort to address these challenges and meet the growing demand for evidence-based practices [1,3,4]. After preliminary work to define the gaps in existing guidelines, a structured consensus workshop was held at ILSI Europe in Brussels in April 2025 to develop expert-driven recommendations for conducting probiotic human studies. Academic researchers, clinicians, and industry professionals contributed to the multidisciplinary discussion.

The outcome of this initiative is the PRObiotic human study Design And REporting (PRODARE) recommendations for improving current practice, focusing specifically on probiotics for oral consumption in foods or dietary supplements.

The PRODARE recommendations represent the core output of this consensus process and are intended to serve as a structured framework to guide the design, conduct, and reporting of human probiotic studies across different study types.

### Challenges in conducting human studies with probiotics

Studies of probiotics in humans are conducted with a range of objectives, from hypothesis generation and feasibility testing to elucidating a mechanism of action or evaluating functionality in real life. In all cases, designing robust and informative human studies with probiotics and probiotic foods and dietary supplements presents a distinct set of challenges. Unlike most nutraceuticals, probiotics are live microorganisms whose effects are strain-specific and depend on factors such as viability, administered dose, and accurate taxonomic identification. These characteristics introduce additional methodological and regulatory challenges that are distinct from those associated with other nutraceuticals.

Existing literature contains many examples of probiotic studies that yield mixed, inconclusive, and even contradictory findings, raising questions about their reliability and the interpretability of outcomes [5]. Although probiotics are often regulated as dietary supplements or functional food ingredients rather than as medicinal products, clinical trials evaluating their effects should adhere to established standards such as Good Clinical Practice. Variability in the application of these standards, together with heterogeneity in strains, doses, study designs, and outcome measures, likely contributes to inconsistencies in study design and outcomes. Furthermore, as probiotics are generally not regulated as medicinal products, their associated health effects are typically not evaluated within the same regulatory frameworks used for disease treatment or prevention claims.

As is common in nutrition research, many intervention studies, including those on probiotics, are conducted in the general population rather than in well-defined patient groups. Probiotic studies often involve individuals experiencing mild or transient conditions such as bloating, constipation, abdominal pain, stress, or upper respiratory tract discomfort during the winter season. These modest health issues make it more difficult to detect and validate beneficial effects. However, studies have also been conducted in patient populations with clearly defined clinical endpoints. Statistically significant and clinically relevant changes in physiological parameters are more likely to be observed in individuals with values outside the normal range due to disease or an at-risk state. In addition, substantial interindividual variability in response, including the presence of nonresponders, is frequently observed in probiotic studies and other nutritional interventions [6]. Such variability is likely linked to differences in baseline health status, medication use, diet, and lifestyle, all of which influence the composition and function of the host microbiome [7]. These challenges are not unique to probiotics but are inherent to nutrition research more broadly.

A small effect size and the absence of validated and approved biomarkers hinder the demonstration of probiotic efficiency [8]. This has implications for the statistical power of a study, as small effect sizes necessitate larger cohorts to increase the probability of observing small effects on given biomarkers. Many probiotic studies are significantly underpowered due to insufficient cohort size. Although study design involves a wide range of methodological, analytical, and statistical considerations, including sample size determination and detailed statistical modeling, these aspects are beyond the scope of the present manuscript, which focuses on study design principles and reporting considerations specific to probiotic interventions.

The fundamental difference between probiotics and most other bioactive substances is that probiotics are living microorganisms. In the design and conduct of human studies with probiotics, it is, therefore, of critical importance to ensure the viability of the probiotic or, as in the case of real-world studies (RWS), to be aware that viability cannot be controlled. Moreover, production, storage, and handling conditions can have a marked influence on live and dead cell count before and during a probiotic trial.

These drawbacks highlight the need to minimize confounding factors in human nutrition intervention trials by ensuring that cohorts are thoroughly characterized and registered. This applies broadly to nutrition and dietary research, including but not limited to probiotics. Variability in individual responses, appropriate endpoint selection, and adequate statistical power are common challenges across all such studies. Establishing agreement on appropriate endpoints and sample sizes is important to ensure that studies are adequately powered to detect meaningful effects and to support comparability across studies. This, in turn, facilitates evidence synthesis and contributes to regulatory evaluation and evidence-based practice. Although well-designed studies with clearly defined endpoints are often necessary for regulatory approval and substantiation of health claims, different study designs may be appropriate depending on the research objective, including hypothesis generation and real-world effectiveness assessment. Study comparability remains essential for meta-analyses and for advancing the overall scientific understanding of probiotics and nutrition interventions [9].

### Purpose and scope

The ILSI Europe workshop was convened to identify a pathway to conducting and reporting fit-for-purpose human trials—across all age groups—with probiotics for dietary supplements or functional foods. This included developing a guide, presented as a decision tree, to facilitate the selection of the appropriate study type and to support evaluations of the need for subsequent investigations.

To establish the framework for the discussion, the workshop set out by exploring the types of study that may be conducted to achieve specific objectives. Exploratory studies, pilot studies, prospective and retrospective cohort studies, randomized controlled clinical trials, and RWS were reviewed to establish a shared understanding of their purpose and methodological requirements in the context of probiotics. Recommendations were then proposed to tailor existing guidelines for each trial type to the probiotic context.

The purpose of this manuscript is not to redefine clinical research methodology, but to integrate probiotic-specific considerations into established human study designs and reporting standards. To this end, the manuscript sets out a methodological framework and practical guidance for probiotic human studies rather than evidence-graded clinical recommendations. The added value of PRODARE lies in systematically incorporating factors such as strain-level characterization, viability, and product stability, which are not explicitly addressed in existing general guidelines.

By focusing solely on probiotic human studies, the workshop presumed that a series of fundamental questions were already resolved and, hence, not in scope. These presumptions are as follows and are in line with the probiotic definition [1,10]:

• The probiotic strain or strains to be investigated are fully characterized and sequenced.
• They have undergone preclinical/safety tests in relation to their intended use in dietary supplements or functional foods.
• Live cell count is stable during the human study.
• Certificates of analysis address strain identity, purity, viability, antibiotic susceptibility, and antimicrobial resistance profiles.
• The human studies in question have ethical approval.

Before and during the workshop, it appeared that the terminology used in descriptions of probiotic clinical trials may be ambiguous. For clarity, each study type is described in the “probiotic human studies” section of this paper. Supplementary Table 1 provides a glossary of additional terms used [[63], [64], [65], [66], [67], [68]]. These elements are considered essential reporting items in human probiotic studies to ensure reproducibility, comparability, and appropriate interpretation of study outcomes.

## Methods: Consensus Development Process

The PRODARE recommendations were developed through a structured, multistep consensus process coordinated by ILSI Europe. An expert group was convened based on recognized expertise in probiotics, clinical research, and human nutrition. The group comprised both academic and industry-based scientists with extensive experience in designing and conducting human intervention studies. Experts represented multiple European countries, reflecting diverse regulatory frameworks, and covered research across different population groups, from infants to adults.

Industry participants were scientists working within research and development departments, with a primary focus on conducting high-quality scientific research, and were not involved in commercial or business development activities. The inclusion of both academic and industry experts was considered a strength, enabling the integration of complementary scientific and practical perspectives.

To minimize potential bias, procedures were implemented in accordance with the ILSI Europe framework for expert group composition. This requires that the expert group include at least an equal number of academic and industry representatives (here: 11 academic and 8 industry participants). The group was chaired by an academic representative.

Consensus development was initiated during a structured workshop held in April 2025. Before the workshop, participants received background materials and predefined questions to guide the discussions. The full list of questions is provided in Supplementary Material 2. During the workshop, existing general guidelines for different study designs [exploratory studies, pilot studies, randomized controlled trials (RCTs), RWS, and observational studies] were reviewed. This was followed by structured discussions to identify considerations specifically relevant to probiotic interventions. The consensus reflects a scientific position developed through structured discussion and collective agreement and is not intended to represent individual or institution-specific viewpoints.

Discussions were guided by predefined questions addressing study design, intervention characterization, outcome selection, dietary and microbiome considerations, and regulatory aspects. Participants were invited to evaluate the presented material, identify gaps, and propose additions or modifications. All discussions were documented by a scientific writer.

After the workshop, a subgroup of authors drafted the manuscript based on the workshop outcomes. The draft was circulated to all contributors for review and comment, and revisions were made iteratively until a final version was agreed upon by all authors. Contributors providing substantial input were included as coauthors.

The consensus reached reflects a scientific agreement on appropriate study designs, markers, and endpoints for probiotic research, with the aim of supporting robust, interpretable, and regulatorily relevant outcomes.

### From probiotic candidate to documented strain—a visual decision tree for probiotic research

The probiotics market has grown rapidly based on a framework of dietary supplement and functional food regulations, which, in some countries, only require manufacturers to confirm the safety of a probiotic product before commercialization [11]. Even in regions such as the European Union, where only approved health claims are permitted, ambiguity in the communication of product benefits can still lead to confusion among clinicians and consumers [12]. In light of recent discoveries about the microbiome, it is clear that probiotic strains have an important role to play in human health. On that basis, there is a pressing need to document their safety and efficacy through well-designed human trials [11].

A systematic approach to building probiotic evidence begins with comprehensive strain characterization and assessments of safety, stability, and mechanisms of action. These are the preconditions of subsequent human studies, which, as previously noted, are outside the scope of this paper. Building on this foundation, the selection of an appropriate human study design should be guided by the specific research objectives and the characteristics of the target population.

**FIGURE 1:**
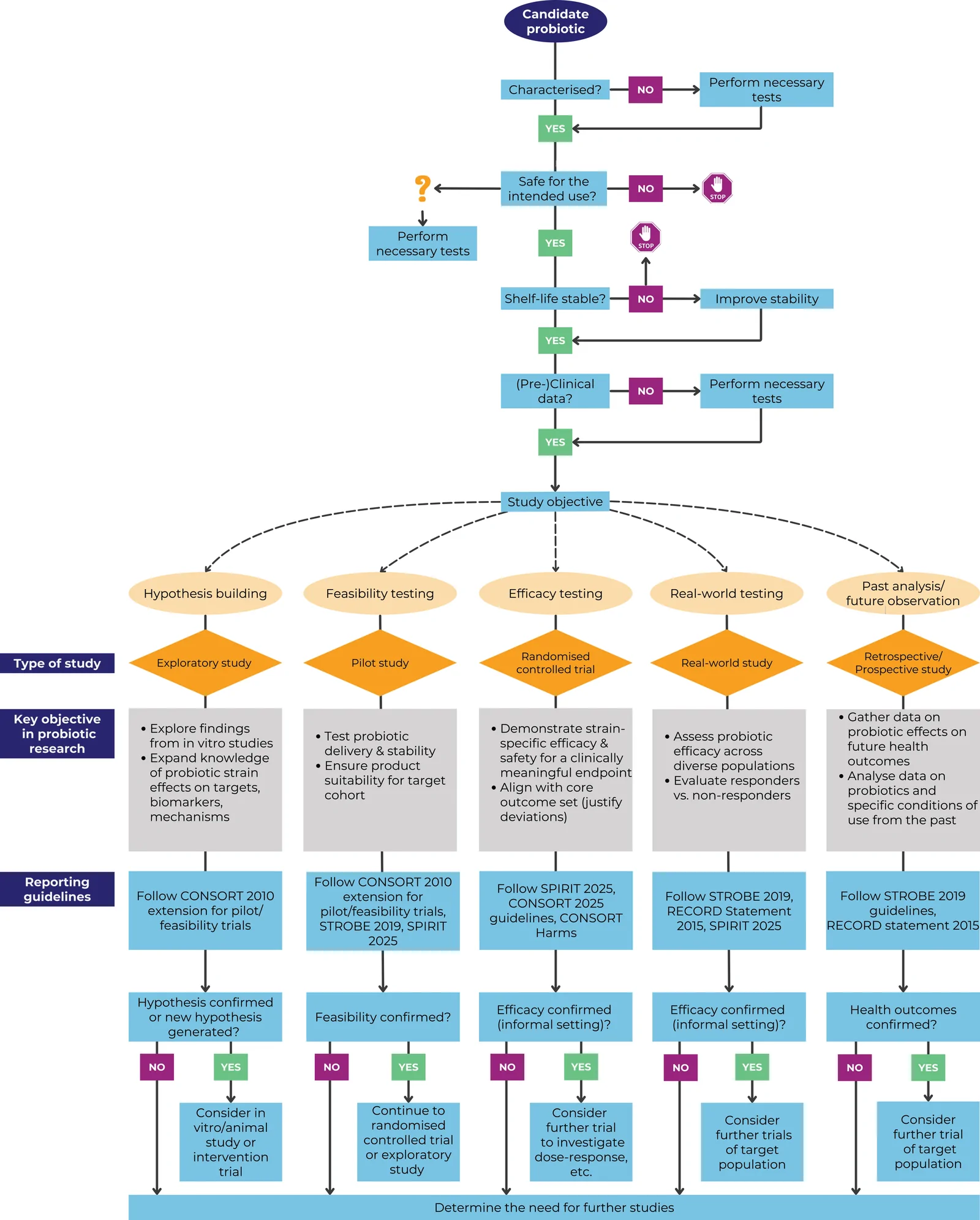
Decision tree for selecting fit-for-purpose probiotic human studies. RECORD, Reporting of Studies Conducted Using Observational Routinely Collected Health Data; SPIRIT, Standard Protocol Items: Recommendations for Interventional Trials.

The decision tree in Figure 1 shows the 5 general objectives of human studies—hypothesis building, feasibility testing, efficacy testing, real-world testing, and past analysis/future observation. The appropriate study type and related guidelines are proposed for each objective. The decision tree represents a consensus-based conceptual framework and is intended as a guidance tool rather than a formally validated instrument. Although RCTs are the reference standard for evaluating the efficacy of interventions in humans, other designs may be more appropriate for questions about mechanisms of action, dosage, adverse events, and impact on health outcomes. To ensure meaningful and comparable outcomes, it is necessary that researchers share the same understanding of study designs and employ a consistent approach to conducting and reporting. The Enhancing the Quality and Transparency of Health Research Network has summarized the well-established reporting guidelines that already exist for most study designs, and which are a good starting point [13]. However, guidance adapted to the specific methodological challenges of probiotic research remains unavailable. The development of such field-specific recommendations for study planning, implementation, and reporting is, therefore, a valuable supplement to existing guidelines.

### The PRODARE recommendations for probiotic human studies

International literature draws attention to a series of general recommendations applicable to probiotic human study protocols to ensure the validity and value of their findings [6,7,14,15]. These are listed below as a prologue to a more detailed discussion of the purpose of and recommendations for individual study types.

### General recommendations

1 *Specify the strain used for the intervention*

Despite a broad consensus that probiotic efficacy is both strain and benefit-specific, many human studies still only report the genus and species of the probiotic used. To distinguish between probiotic products and ensure reliable efficacy analyses, strain designations must be clearly reported [14,15].

2 *Record the diet, lifestyle, and age of the study population*

The varying dietary habits and lifestyles of individuals have a known effect on the gut microbiome and, subsequently, on probiotic efficacy. In some cases, such variations may differentiate responders from nonresponders to a probiotic intervention [7]. For this reason, recording and reporting diet, lifestyle, and other influencing factors, such as the age, sex, nutritional and health status, or medication use of study participants, are strongly recommended. Detailed guidance on how dietary factors should be addressed in probiotic and prebiotic clinical trials has been summarized elsewhere and can support the development of robust and interpretable study protocols [7]. Depending on the study objective, minimizing variations through appropriate inclusion/exclusion criteria before intervention and stipulating how and when the probiotic should be consumed may enhance the reliability and interpretability of the results. The Strengthening the Organization and Reporting of Microbiome Studies reporting guidelines are recommended for studies that focus on microbiome-related endpoints [16].

3 *Determine the washout period before and after the intervention*

This refers to the period in which participants refrain from consuming foods or supplements that contain, for example, probiotics and other possible confounders before or between interventions [17,18]. If fermented foods that contain live microbes (not probiotics) are part of the staple diet, it is recommended to record this. Medication such as antibiotics should also be avoided, unless they are being investigated as a confounding factor. In populations where medication is commonly used (e.g., mature adults, people with obesity, etc.), stable use is required, and it is recommended to record the use. Alignment of washout periods is another way to minimize variations between individual study participants.

4 *Implement risk management measures to prevent cross-contamination*

When conducting a study in an institutional setting, such as a hospital or care home, researchers should assess the probability of cross-contamination between areas and residents while the probiotic is in use on site. This can both pose safety concerns for high-risk subjects and potentially affect study outcomes [19,20]. Identified concerns should be managed and reported.

5 *Align definitions of measurable endpoints*

Systematic reviews and meta-analyses are best served by clinical trials that measure the same endpoints. However, the use of differently defined outcomes to evaluate efficacy is common practice—for example, the duration of diarrhea may be defined as the time to the first normal stool or the last diarrhea [21]. Establishing and using core outcome sets (COSs) is, therefore, recommended to ensure consistent endpoint definitions, improve comparability across studies, and strengthen the overall evidence base [22].

6 *Standardize reporting on probiotic dosage and live cell count*

Probiotics are defined as “live microorganisms that, when administered in adequate amounts, confer a health benefit on the host” [1]. Yet a comparison of registered probiotic trials has revealed that dosage is sometimes reported incorrectly and that there is a wide variation in the CFU administered daily and the duration of consumption [14]. Accurate declaration of dosage is invaluable when assessing probiotic efficiency. Manufacturer storage recommendations should be followed to standardize and preserve CFU before and during a study. In the case of freeze-dried probiotics, cell count is best maintained through storage in cool, dry conditions. Also, fresh fermented products are best stored refrigerated. If other measures of quantification are used, for example, active fluorescent units or molecular methods, information about the measuring technique should be disclosed.

7 *Register to secure publication and transparency*

Preregistration of studies in a recognized clinical trial registry is the best practice to qualify for future publication in a scientific journal [14]. From such registries, researchers can gain important insights into available evidence from previous trials, avoid duplication, and identify collaboration partners. The WHO offers a collated database of international registries at its Clinical Trials Search Portal [23]. Many regional and national registries also exist.

### Probiotic human studies—outline and recommendations

The ILSI Europe workshop identified 5 types of human studies relevant to probiotic research. These are outlined in the following, along with recommendations for conducting and reporting on each study type. Although suggestions are provided for the design and implementation of different study types, these are not intended to be prescriptive or restrictive. Depending on the research question, multiple study designs may be combined or applied iteratively, and additional methodological approaches may be considered where appropriate.

### Exploratory studies

Exploratory studies can be first-in-human studies, performed early in phase 1 to evaluate the effect of one or more interventions or build hypotheses, possibly including a placebo or other control, on health-related or behavioral outcomes. In the absence of formal guidelines for exploratory studies with probiotics, they are typically conducted with a limited number of participants [24].

Often confused with pilot studies, which test the premises for a larger randomized controlled trial, exploratory studies may have no therapeutic or diagnostic goal [25]. Instead, the role of exploratory studies is to explore a hypothesis or question, typically based on limited, usually in vitro or in vivo evidence, but may also be based on existing clinical evidence. Objectives may be to identify new research endpoints and/or biomarkers, to create a theoretical understanding of a potential health benefit, and to inform sample size calculations [24]. By providing preliminary estimates of effect size, variability, and feasibility parameters, exploratory studies help refine statistical assumptions. In this way, they generate evidence that ensures further, more extensive clinical investigations are adequately powered [26].

The following recommendations (Table 1 [27]) are an attempt to build consensus about the definition of exploratory studies in terms of their objectives, design, and conduct, and to provide guidance on the evidence needed to inform decision-making about further studies [28].

**TABLE 1:** Recommendations to clarify and optimize probiotic exploratory studies

Definition	Exploratory studies can be first-in-human trials that: • Evaluate the effect or safety of one or more interventions • Build hypotheses on benefit-related outcomes
Key objectives in probiotic research	• Answer specific questions arising from existing in vitro, animal, or clinical evidence • Expand knowledge about the effect of specific probiotic strains on identified targets, biomarkers, and mechanisms • Enable refinement of future power calculations
Design and implementation	• Follow the general recommendations for probiotic human studies given above. The study design can be flexible. • Specific recommendations for probiotic exploratory studies: ○ Define case-by-case criteria to inform poststudy decision-making on whether and how to proceed with further research. Key considerations include: ⁃ The suitability of the strain or strain combination ⁃ The suitability of the dose ⁃ Probiotic stability during the trial ⁃ Product acceptance by participants ⁃ Reports of product-related adverse events ⁃ Possibility of intellectual property protection
Reporting guidelines	• CONSORT extension to randomized pilot and feasibility trials [27]
Potential follow-on probiotic human studies	• If the study has indicated a valuable hypothesis: ○ Check for additional information from existing *in vitro*, *in vivo*, and human studies ○ Consider testing the hypothesis in vitro or in vivo, conduct a pilot study, or plan a randomized controlled trial.

### Pilot studies

Pilot studies provide critical insights about the feasibility of study protocols and address specific areas of uncertainty, thereby guiding the design of subsequent, larger-scale clinical trials [27]. Objectives may include the development and testing of implementation strategies and the evaluation of trial methods [29]. In a probiotic context, recruitment strategies, intervention delivery, and compliance are particularly relevant, along with investigations of the methodology for measuring study parameters and outcomes [30,31].

A common misconception is that pilot studies are equivalent to exploratory studies and can be used to test a hypothesis or assess safety or tolerability. Pilot studies are usually underpowered for such purposes, and caution should be exercised to avoid misinterpretation of results [30,32].

On the contrary, pilot studies require a well-developed research question with specified aims and a rationale to determine whether a future trial can be done, if it should be done, and, subsequently, how it should be conducted [27]. The means of reaching these conclusions should be integrated into the study design as a key objective. Requirements regarding the sample size and compliance with the intervention should also be part of the study’s statistical plan [31].

Multiple randomized or nonrandomized pilot studies may be carried out for feasibility testing [31]. Using the findings from a high-quality pilot study, future RCTs have a greater likelihood of generating useful data. Nonrandomized pilot studies may also be used to inform large-scale cohort studies [33]. Proposed recommendations to clarify and optimize probiotic pilot studies are presented in Table 2 [28,33,34].

**TABLE 2:** Recommendations to clarify and optimize probiotic pilot studies

Definition	Pilot studies are small-scale trials that: • Investigate the feasibility of study designs • Clarify specific areas of uncertainty • Inform subsequent, larger clinical trials
Key objectives in probiotic research	• Test probiotic delivery logistics and stability during the intervention • Ensure the probiotic product is appropriate for the target cohort • Assess any associated adverse events
Design and implementation	• Follow the general recommendations for probiotic human studies given above • Specific recommendations for probiotic pilot studies: ○ Stipulate the key questions that should be answered before a larger investigation. Key considerations include: ⁃ Probiotic delivery logistics ⁃ Selection of target population ⁃ Size of the study group ⁃ Intervention matrix ⁃ Data management and analysis plan ⁃ Feasibility of approaches to measuring outcomes
Reporting guidelines	• CONSORT extension to randomized pilot and feasibility trials [28] • STROBE 2019 guideline, including an adaptable reporting checklist for nonrandomized studies [33] • SPIRIT 2025 guideline [34]
Potential follow-on probiotic human studies	• RCT after verification of the intervention methods and logistics • Additional pilot studies of outstanding questions before a future RCT or exploratory study

Abbreviations: RCT, randomized controlled trial; SPIRIT, Standard Protocol Items: Recommendations for Interventional Trials.

### Randomized controlled trials

RCTs evaluate the effectiveness or safety of a health-related intervention. Recognized as the reference standard for clinical research, they are widely seen as a source of high-quality evidence, surpassed only by systematic reviews or meta-analyses of RCTs [5]. Well-conducted RCTs are characterized by their minimization of bias and documentation of cause-and-effect relationships.

Within the field of probiotic research, RCTs are widely initiated to investigate the effect of specific strains in relation to, for example, gastrointestinal health, immune function, brain health, allergies, and eczema [[35], [36], [37], [38], [39]]. The choice of studies is usually based on existing (pre)clinical evidence. However, even well-structured probiotic RCTs often produce ambiguous results [5]. Variability of strains, formulations, and doses, the placebo effect on subjective outcomes, and the complex interactions that occur with the gut microbiome are among the challenges. The lack of validated probiotic biomarkers also limits the ability of RCTs to document cause-and-effect mechanisms—a major hurdle when seeking authority approval for probiotic health claims. Where RCTs are of longer duration, it is particularly important to monitor and report product viability.

Internationally agreed COSs, as facilitated by the Core Outcome Measures in Effectiveness Trials (COMET) Initiative [22], are contributing to a standardization of RCTs, improving their quality and consistency, and supporting decision-making by clinicians [[40], [41], [42]]. Developed for a specific disease or health condition, COSs cover the minimum endpoints relevant to routine clinical practice that should be measured and reported, including benefits and harms. In this way, they both improve the likelihood that appropriate outcomes are measured and maximize the potential for comparing results across trials. These should be considered when designing an RCT for probiotics.

For designing and conducting a fruitful RCT with probiotics, researchers can confidently follow existing guidelines [9,43].

Proposed recommendations to clarify and optimize probiotic RCTs can be found in Table 3 [7,22,34,[44], [45], [46]].

**TABLE 3:** Recommendations to clarify and optimize probiotic randomized controlled trials

Definition	RCTs are recognized as reference standard trials that: • Evaluate the effectiveness or safety of a health-related intervention
Key objectives in probiotic research	• Demonstrate strain-specific efficacy and safety for a prespecified, clinically meaningful endpoint [44]. Considering existing preclinical and clinical evidence
Design and implementation	• Follow the general recommendations for probiotic human studies given above • Specific recommendations for probiotic RCTs: ○ Build investigations of appropriate available core outcome sets into the design [22] ○ Ensure proper blinding of sensory characteristics (taste, smell, appearance) in the probiotic test product and placebo ○ Monitor participant adherence and intake ○ Monitor product quality and contaminants (absence of transferable antimicrobial resistance) ○ Manage probiotic safety issues (strain-matched bacteremia/fungaemia and risks for vulnerable or immunocompromised individuals)
Reporting guidelines	• The SPIRIT 2025 guideline [34] • CONSORT 2025 guidelines [45] • CONSORT Harms [46]
Potential follow-on probiotic human studies	• Dose–response trials to find the minimum effective dose • Responder-enrichment studies to focus on subjects most likely to benefit based on their microbiome or diet • Diet–probiotic interaction trials to test how different diets change the effect of probiotics [7] • Postmarketing safety registries to track rare side-effects when probiotics are widely used

Abbreviations: RCT, randomized controlled trial; SPIRIT, Standard Protocol Items: Recommendations for Interventional Trials.

### RWS

RWS are increasingly recognized as credible, publishable science, providing insights into how probiotics perform in everyday practice outside a traditional, controlled clinical setting, thereby using existing (pre)clinical data as a starting point. By accounting for the natural variability in the environment, human behavior, and health status, RWS provide insights into probiotic efficacy in diverse populations and may contribute to a mapping of responders compared with nonresponders [47].

Data used for RWS can be generated from a variety of methodologies, such as biological samples, electronic health records, patient registries, interviews, quality of life surveys, and other types of questionnaires and technology [48,49]. In this way, RWS evaluate both the objective and subjective effectiveness of interventions. Regardless of the informal environment in which they are conducted, a detailed study protocol with a clear research question is a requirement. RWS must also be ethically sound with respect to data protection, subject privacy, and transparency [50,51]. Because of the large data volumes that many RWS generate, powerful data processing and analysis techniques may be required [52].

The use of RWS has, until now, largely been driven by the pharmaceutical sector. Although no guidelines have been developed specifically for health interventions via the diet, the general points in RWS guidelines for safety assessment of medicines are a good starting point for planning, designing, and conducting RWS of probiotic efficacy [51,53]. It should be noted that there can be limitations to RWS, such as *1*) susceptibility to various forms of bias (e.g., selection bias, confounding, and information bias), *2*) variability and potential limitations in data quality and standardization across real-world data sources, and *3*) the challenges associated with drawing causal inferences in nonrandomized settings. These limitations are particularly relevant in probiotic research, where heterogeneity in strains, dosing regimens, patient populations, and outcome measures can complicate interpretation. Accordingly, we contrast these challenges with the strengths of RCTs and emphasize the complementary roles of both or multiple approaches for efficacy determination.

Proposed recommendations to clarify and optimize probiotic RWS can be found in Table 4 [33,34,54,55].

**TABLE 4:** Recommendations to clarify and optimize probiotic real-world studies

Definition	RWS may be conducted before or after an RCT to: • Gather evidence about the practical performance of a medical or dietary intervention • Evaluate objective and subjective effectiveness outside a clinical setting
Key objectives in probiotic research	• Provide insights into probiotic efficacy/health outcomes in diverse populations under real-world conditions, considering existing preclinical and clinical evidence. • Evaluate responders compared with nonresponders to probiotic intervention
Design and implementation	• Follow the general recommendations for probiotic human studies given above • Specific recommendations for probiotic RWS: ○ Design detailed study protocols, specifying methods and interventions ○ Specify methods for selecting and analyzing data that is fit-for-purpose in respect of the research question ○ Include a plan for capturing data about the probiotic intervention, including storage, method and duration of consumption, compliance, purchase location and expiry date ○ Include a plan for capturing data about the participants, including lifestyle and health status ○ Plan for handling data anomalies, such as missing data
Reporting guidelines	• STROBE 2019 guideline [33] • RECORD statement 2015 [54] • SPIRIT 2025 guidelines [34] • ISPOR and ISPE Task Force recommendations [55] When conducting a study for regulatory purposes, consult, if possible, with regulatory bodies before execution to ensure compliance with ethical and scientific standards in the specific territories
Potential follow-on probiotic human studies	• Exploratory, pilot, or randomized controlled trial if study findings provide the basis for new hypotheses • Further real-world investigation in other territories

Abbreviations: ISPE, International Society for Pharmacoepidemiology; ISPOR, International Society for Pharmacoeconomics and Outcomes Research; RCT, randomized controlled trial; RECORD, Reporting of Studies Conducted Using Observational Routinely Collected Health Data; RWS, real-world studies; SPIRIT, Standard Protocol Items: Recommendations for Interventional Trials.

### Observational studies: prospective and retrospective cohort designs

Prospective and retrospective studies are observational cohort studies that aim to link outcomes with exposure to a health intervention over time [56]. Though both may draw on real-world data collected from everyday healthcare settings and additional (pre)clinical data, they are not to be confused with RWS.

In prospective studies, researchers follow participants over time to observe how exposure to treatment or lifestyle change may impact future outcomes [56]. Prospective studies allow greater control over data collection, standardization, and follow-up [57].

Retrospective studies, to the contrary, use existing or historical data to investigate the potential causes of or risk factors for a condition that occurred in the past. Although often faster and more cost-efficient than prospective studies, they are more susceptible to bias and confounding factors due to a lack of control over data quality and completeness.

To date, few cohort studies of either kind have investigated health interventions with probiotics [[58], [59], [60], [61]], and there are, consequently, no existing guidelines for the probiotic field. Some general guidance is available; however, as noted in the recommendations below [54,62]. A checklist for probiotic cohort study designs, conduct, and data analysis would be a valuable tool to build consensus around a common approach.

Proposed recommendations to clarify and optimize probiotic prospective and retrospective studies can be found in Table 5 [33,34].

**TABLE 5:** Recommendations to clarify and optimize probiotic prospective/retrospective studies

Definition	Prospective and retrospective studies are observational cohort studies that: • Investigate the effects of a health intervention in a cohort over time
Key objectives in probiotic research	• Gather data about the effect of a probiotic dietary supplement or food on future health outcomes, considering existing preclinical and clinical evidence. • Analyze data on probiotics and specific conditions of use from the past
Design and implementation	• Follow the general recommendations for probiotic human studies given above • Specific recommendations for probiotic prospective/retrospective studies: ○ Specify research endpoints and biomarkers of interest ○ Identify the cohort of interest and the rationale for selection ○ Include a detailed plan for data collection and analysis
Reporting guidelines	• STROBE 2019 guideline [33] • RECORD statement 2015 [54]
Potential follow-on human studies	• Exploratory, pilot, or randomized controlled trial if study findings provide the basis for new hypotheses

Abbreviations: RECORD, Reporting of Studies Conducted Using Observational Routinely Collected Health Data.

## Discussion

The PRODARE recommendations reflect the expert consensus from a cross-disciplinary workshop convened by the Probiotics Task Force of ILSI Europe to promote a more standardized approach to probiotic human studies.

By specifying the methodological requirements of human studies involving probiotics, the recommendations provide a framework for improving the reliability and comparability of study designs and strengthening the relevance and rigor of their findings. The ultimate aim is to advance knowledge about how specific probiotic strains modulate the human microbiome and contribute to health benefits.

Although existing reporting guidelines provide a solid foundation for human study reporting, they do not address challenges unique to probiotics as live microorganisms with strain-specific effects. Minor differences in strain identity and viability can have a substantial influence on outcomes. Clear documentation of strain designation, dose, formulation, and administration is, therefore, essential to enable reproducibility and cross-study comparison. PRODARE complements general reporting standards by emphasizing the need to address these probiotic-specific considerations in alignment with established best practices.

At present, many questions remain about the health impacts of consuming individual probiotic strains in foods and dietary supplements. Regulatory evaluations have underscored this uncertainty. The European Food Safety Authority, for example, has not approved any probiotic-related health claims to date due to insufficiently substantiated evidence. In contrast, other regulatory bodies, including Health Canada, the Brazilian National Health Surveillance Agency, and the Australian Therapeutic Goods Administration, have authorized certain claims, whereas in the United States, structure-function claims are permitted under a different regulatory framework. PRODARE provides a framework for standardizing the design, conduct, and reporting of human probiotic studies. Such a standardized approach can advance scientific understanding and lay the foundation for translating probiotic research into evidence-based clinical and public health applications.

The ILSI Europe workshop that initiated this paper highlighted several fundamental gaps in current knowledge, including the need for a better understanding of interactions between probiotics, other dietary components, and the existing microorganisms in the gut. This raises a broader and more critical question concerning the identification of measurable outcomes that extend beyond merely observing changes in microbiota composition. In this context, the need for validated biomarkers that capture the probiotic effect is evident.

Moving forward, coordinated efforts by academic, clinical, and industry stakeholders are necessary to pursue the implementation of the PRODARE framework. Pilot applications of the decision tree are recommended in diverse settings to validate its utility and refine the guidance based on practical experience. The decision tree currently represents a consensus-based conceptual framework designed by experts in the field and has not yet been formally validated in empirical studies. Collaborative platforms such as ILSI Europe and international microbiome consortia are encouraged to foster consensus across regions. Further investment in shared infrastructures—including repositories for probiotic strain data, standardized outcome definitions, and validated biomarkers—is essential to support evidence synthesis and transparency.

In the longer term, alignment of PRODARE with existing international initiatives, such as the COMET Initiative, is recommended to promote formal recognition of probiotic-specific methodological guidance and accelerate the translation of research into evidence-based practice and policy.

In conclusion, a structured approach to the design, conduct, and reporting of probiotic human studies is imperative to generate reliable evidence and guide future clinical practice. By complementing existing reporting frameworks and addressing the specific challenges of probiotics, PRODARE offers practical recommendations for more reliable, interpretable, and impactful research in this field.

## Author contributions

The authors’ responsibilities were as follows – ACO, NV, JL: conceived the idea; HS, MB, GC, AC, JL, PSM, CAvL-B, GW, NV, ACO: prepared the workshop, conducted the literature search, and contributed to data curation; and all authors: critically revised the manuscript for important intellectual content, contributed to writing, approved the final version, and agreed to be accountable for all aspects of the work.

## Declaration of Generative AI and AI-assisted Technologies in the Writing Process

The authors declare that no generative AI or AI-assisted technologies were used in the writing of this manuscript.

## Funding

This work was conducted by an expert group (EG) of the European branch of the International Life Sciences Institute (ILSI) Europe. According to ILSI Europe policies, the EG is composed of ≥50% external nonindustry members. The complete composition of the EG can be found on the ILSI Europe website (https://ilsi.eu/scientific-activities/nutrition/probiotics/). Experts are not paid for the time spent on this work. However, nonindustry members were offered support for travel and accommodation costs from the above-mentioned task forces when attending workshops/meetings to discuss the manuscript. Journalist and communication consultant Cath Mersh received funding for her writing services from the above-mentioned Task Force.

## Conflict of interest

HS serves as a board member of the International Scientific Association for Probiotics and Prebiotics, an unpaid and voluntary position. She has received honoraria and/or research funding for work as a clinical investigator, advisory board member, consultant, and speaker for companies manufacturing probiotics and/or prebiotics. CHPvdA has participated as a consultant or speaker for Nestlé Nutrition Institute and Nutricia Early Life Nutrition. Reimbursements used as research funds within the hospital. MCC has received speaker honoraria and/or been involved in research funding from some companies that manufacture probiotics and/or prebiotics. JL, CAvL-B, CF, DPP, NV, and ACO work full time for H & H Group, Yili Innovation Center Europe, Cargill, Danone, Yakult, and IFF, respectively. These companies were not involved in carrying out this research. All other authors report no conflicts of interest.
