# Supplementary material for: Probiotic Human Study Design and Reporting (PRODARE): Recommendations to Improve Research Practice
Source: Adv Nutr. 2026 Jun 2;17(8):100668. doi: 10.1016/j.advnut.2026.100668 (PMC13494636; doi:10.1016/j.advnut.2026.100668)
Supplement: Supplementary file 1 [file mmc1.docx]

**Supplementary Table 1:** Glossary of terms used in this paper

| **Term** | **Intended definition in this paper** |
| --- | --- |
| Core outcome set | An agreed standardized set of outcomes that should be measured and reported, as a minimum, in all clinical trials in specific areas of health or healthcare (21). |
| Efficacy testing | The ability of a given probiotic strain to have a statistically and biologically proven effect on a health condition or disease and/or quality of life and well-being (63). |
| Feasibility testing | A preliminary investigation to assess whether an intervention is appropriate for further testing, for example in a larger clinical trial (64. |
| Human study | Research involving human participants, including surveys, observational studies and interventions. It is not necessarily focused on medical or health-related outcomes (65). |
| Hypothesis building | The gathering and analysis of observational data for developing initial explanations or predictions (66). |
| Probiotic stability | The ability of probiotics to remain viable when stored according to the manufacturer’s specified storage conditions (67). This does not include viability during gastrointestinal transit. |
| Real-world testing | Evaluation of a product’s performance and functionality outside a controlled lab setting, involving real users in everyday conditions (68). |

**Supplement Material 2:** Workshop questions and discussion framework

**General question on the on presentations**

- Do you agree with what has been presented?
- Did we miss any important points, or is there something you would like to add?

**General discussion and recommendations**

- What would you change or include in the existing recommendations?

**Probiotic-specific considerations across study types**

- For each study type, participants were asked to consider specific recommendations for planning, conducting, and reporting probiotic interventions:
  - Exploratory studies – evaluating health-related outcomes prospectively
  - Pilot studies – small-scale studies assessing trial feasibility
  - Randomized controlled clinical trials (RCTs) – gold standard for assessing intervention effectiveness
  - Real-world studies – evaluating probiotic interventions in non-experimental settings
  - Retrospective/prospective observational studies – based on real-world probiotic usage

**Key focus areas for recommendations**

**Study design considerations**

- How can diet and lifestyle be controlled to minimize confounding effects?
- Should participants using specific medications (e.g., antibiotics, proton pump inhibitors) be included or excluded?
- What are the implications for populations with high medication use (e.g., elderly)?
- What is the appropriate washout period for antibiotics or probiotics?
- How does the washout period influence study design (e.g., crossover vs. parallel groups)?

**Intervention and outcome measurement**

- What is the appropriate placebo or comparator for probiotic studies?
- Should gut microbiota changes and host biomarkers of effect/function be measured?
- How do we define and measure a meaningful effect in a healthy population?
- How should probiotic product documentation be standardized (strain(s), dose, formulation, viability, shelf-life, etc.)?
- How can appropriate blinding be ensured when probiotics have sensory characteristics (taste, smell)?
- Should participants be stratified based on baseline microbiota composition, age, BMI, or habitual diet?
- How should responders and non-responders be defined and analysed in probiotic RCTs?

**Dietary and microbiome considerations**

- How can diet be harmonised before intervention to reduce variability?
- Should probiotic trials be avoided during holiday periods due to dietary fluctuations?
- How detailed should prebiotic/probiotic formulation reporting be?
- What are best practices for recording intake (dose, timing, meals, food interactions)?
- How should dietary intake be assessed at screening, baseline, and end of study?
- How can microbiome-relevant dietary exposures be systematically recorded?
- Should dietary assessment follow a hierarchical approach (nutrient level, food level, dietary pattern)?
- What are optimal approaches to microbiome analyses in probiotic trials?
- Should a dietitian or nutritionist be included in probiotic clinical trial teams?

**Additional considerations**

- How does delivery format (capsule vs. fermented dairy) influence outcomes?
- How do lifestyle, diet, and ethnicity affect probiotic activity?
- What are implications for global studies and regulatory requirements?
- What analyses are required to characterize a probiotic strain?
- What regulatory bodies require notification, and under what conditions?
- What preclinical tests are necessary before human studies to demonstrate probiotic efficacy?
- What are the key recommendations for reporting human probiotic studies?
